# Supplementary material for: Molecular and clinical analysis of 27 German patients with Leber congenital amaurosis
Source: PLoS One. 2018 Dec 21;13(12):e0205380. doi: 10.1371/journal.pone.0205380 (PMC6303042; doi:10.1371/journal.pone.0205380)
Supplement: S1 Table — Genes associated with LCA according to RetNet (https://sph.uth.edu/Retnet/) are shown in bold. (DOCX) [file pone.0205380.s001.docx]

**S1 Table. List of genes included in the MIP panel.** Genes associated with LCA according to RetNet (https://sph.uth.edu/Retnet/) are shown in bold.

*ABCA4, ABHD12, ADAM9, ADAMTS18,* ***AIPL1****, ARL2BP, ARL6, BBS1, BBS2, BEST1, C21orf2, C2orf71, C8orf37, CA4,* ***CABP4****, CACNA1F, CACNA2D4, CDHR1,* ***CEP290****, CERKL, CHM, CLN3, CLRN1, CNGA1, CNGA3, CNGB1, CNGB3, CNNM4,* ***CRB1****,* ***CRX****, CYP4V2, DHDDS,* ***DTHD1****, EMC1, EYS, FAM161A, FSCN2, GPR125, GUCA1A, GUCA1B,* ***GUCY2D****, IDH3B,* ***IMPDH1****, IMPG1, IMPG2,* ***IQCB1****, ITM2B,* ***KCNJ13****, KCNV2, KIAA1549, KLHL7,* ***LCA5****,* ***LRAT****, MAK,* ***MERTK****, MFRP, NEK2,* ***NMNAT1****, NR2E3, NRL, OFD1,* ***OTX2****, PDE6A, PDE6B, PDE6C, PDE6G, PDE6H, PDZD7, PITPNM3, PRCD, PROM1, PRPF3, PRPF31, PRPF6, PRPF8,* ***PRPH2****, RAB28, RAX2, RBP3,* ***RD3****,* ***RDH12****, RDH5, RGR, RHO, RIMS1, RLBP1, ROM1, RP1, RP1L1, RP2, RP9,* ***RPE65****, RPGR,* ***RPGRIP1****, SAG, SEMA4A, SNRNP200,* ***SPATA7****, TOPORS, TTC8,* ***TULP1****, UNC119, USH1C, USH2A, VCAN, WDR19, ZNF408, ZNF513*
